# Apoptosis in HEp-2 cells infected with *Ureaplasma diversum*

**DOI:** 10.1186/0717-6287-47-38

**Published:** 2014-09-04

**Authors:** Aline Teixeira Amorim, Lucas Miranda Marques, Angelita Maria Oliveira Gusmão Santos, Hellen Braga Martins, Maysa Santos Barbosa, Izadora Souza Rezende, Ewerton Ferraz Andrade, Guilherme Barreto Campos, Tássia Neves Lobão, Beatriz Araujo Cortez, Telma Alvez Monezi, Glaucia Maria Machado-Santelli, Jorge Timenetsky

**Affiliations:** Instituto Multidisciplinar em Saúde, Núcleo de Tecnologia em Saúde, Universidade Federal da Bahia, Vitória da Conquista, Brazil; Instituto de Ciências Biomédicas, Departamento de Microbiologia, Universidade de São Paulo, São Paulo, Brazil; Instituto de Ciências Biomédicas, Departamento de Biologia Celular e do Desenvolvimento, Universidade de São Paulo, São Paulo, Brazil

**Keywords:** *Ureaplasma diversum*, Invasion, Apoptosis, HEp-2 cells

## Abstract

**Background:**

Bacterial pathogens have many strategies for infecting and persisting in host cells. Adhesion, invasion and intracellular life are important features in the biology of mollicutes. The intracellular location of *Ureaplasma diversum* may trigger disturbances in the host cell. This includes activation or inhibition of pro and anti-apoptotic factors, which facilitate the development of host damage. The aim of the present study was to associate *U. diversum* infection in HEp-2 cells and apoptosis induction. Cells were infected for 72hs with four *U. diversum* clinical isolates and an ATCC strain. The *U. diversum* invasion was analyzed by Confocal Laser Scanning Microscopy and gentamicin invasion assay. The apoptosis was evaluated using pro-apoptotic and anti-apoptotic gene expression, and FITC Annexin V/Dead Cell Apoptosis Kit.

**Results:**

The number of internalized ureaplasma in HEp-2 cells increased significantly throughout the infection. The flow cytometry analysis with fluorochromes to detect membrane depolarization and gene expression for caspase 2, 3 and 9 increased in infected cells after 24 hours. However, after 72 hours a considerable decrease of apoptotic cells was observed.

**Conclusions:**

The data suggests that apoptosis may be initially induced by some isolates in association with HEp-2 cells, but over time, there was no evidence of apoptosis in the presence of ureaplasma and HEp-2 cells. The initial increase and then decrease in apoptosis could be related to bacterial pathogen-associated molecular pattern (PAMPS). Moreover, the isolates of *U. diversum* presented differences in the studied parameters for apoptosis. It was also observed that the amount of microorganisms was not proportional to the induction of apoptosis in HEp-2 cells.

## Background

Apoptosis is characterized by a series of dramatic perturbations in the cellular architecture that contribute not only to cell death, but also prepare cells for removal by phagocytes and prevent unwanted immune responses [1]. However, pathological cell death is not regulated, causing disturbances in a tissue, organ and the whole organism [2].

Apoptosis is natural, but may be activated or accelerated, for instance, by toxic or mutagenic agents, and events blocking the spread of infection [3]. The apoptosis of a cell involves self-signaling that causes self-death. This process could be initiated by TNF-α receptors, Fas a ligand protein and its receptor [4] and the intrinsic pathway. These distinct pathways may converge into a single path and activate caspases [1]. Caspases are normally present in healthy cells as inactive precursor enzymes (zymogens) with little or no protease activity. However, all stimuli that trigger apoptosis seem to do so by initiating events that culminate in caspase activation, albeit in somewhat different ways. To date, three main routes to apoptosis associated caspase activation have been firmly established in mammals [1].

Apoptosis can also be induced by an infectious agent, which interferes with the activation or the inhibition of the apoptotic cascade [5–7, 3, 8]. This inhibition blocks the replication of the infectious agent or allows it to escape from the host immune response, as occurs in intracellular microorganisms such as *Chlamydia trachomatis, Neisseria meningitidis* and some viruses [9, 10, 3, 6]. In activation, microbial toxins can interact with the signaling pathways of the host cell death, as occurs with *Leptospira interrogans, Shigella dysenteriae* and *Escherichia coli* [7, 3, 6, 8].

Microorganisms of the class *Mollicutes* such as *Mycoplasma bovis, Mycoplasma penetrans* and *Ureaplasma urealyticum* also possess virulence mechanisms involving apoptosis of host cells [11–13]. These microbial species are also closely related in the development of urogenital pathologies in humans or animals.

*U. diversum* is a facultative intracellular microbe, i.e. it can dwell on the surface of host cells as well as inside [14]. Fish et al. [15] showed that *U. diversum* could be isolated from the genital tract of cattle, being reported as a major cause of genital disorders in these animals [16–18]. In fact this ureaplasma is related to granular vulvitis, low-sperm motility, infertility and abortion in bovines [19, 15, 20, 16]. Nevertheless, little is known about the virulence and pathogenic mechanisms of this mollicute. Studies with human origin ureaplasmas have suggested that once inside the cell, these bacteria can induce cytopathic effects [21] due to production of proteases, nucleases and phospholipases, whose superoxide radicals can lead to a clastogenic effect [20, 22].

Many microorganisms possess several virulence factors that could affect the stability of host cells and may result in death. Marques et al. [22] analyzed *U. diversum* infection for 12 hours and observed that these microorganisms were detected inside the cells after one minute, and after three hours, the invasion of the ureaplasmas surrounded the nuclear region but were not observed inside the nuclei. The present study aimed to evaluate the apoptosis of HEp-2 cells experimentally infected with *U. diversum* during 72 hours of infection. Cells were infected and analyzed by Confocal Laser Scanning Microscopy and gentamicin invasion assay to confirm the invasion process. The apoptosis of cells was evaluated for their caspase gene expression and by flow cytometry methodologies. This may initially facilitate better understanding of the interaction of bovine origin ureaplasma with the apoptosis of HEp-2 cells, being the most used cell lineage in host-parasite studies with *Mollicutes*.

## Results

### Invasion assays

#### Confocal laser scanning microscopy

**Figure 1:**
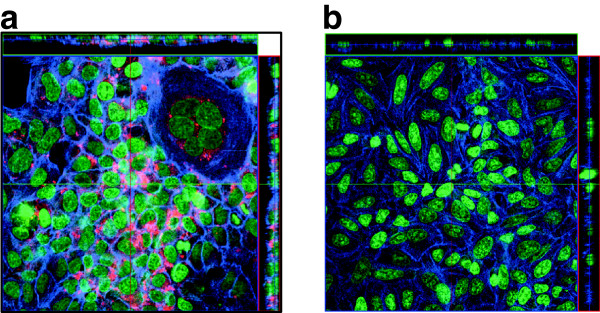
**LSCM optical sections showing internalization of U. diversum in Hep-2 cells and control group after 72 hours. a**. Infection of the U. diversum strain ATCC 49782 in Hep-2 cells. Ureaplasmas were labeled with Vibrant Dil (in red), Hep-2 actin filaments stained with phalloidin-FITC (in blue) and Hep-2 nuclei stained with TO-PRO-3 (in green). **b**. LSCM optical sections showing Hep-2 cells not infected with U. diversum (control group).

In order to monitor and compare the capacity of several ureaplasma strains to invade HEp-2 cells, we used Confocal Laser Scanning Microcopy. Throughout the studied infection period, we observed that most ureaplasma showed perinuclear localization, though we did not observe invasion of this compartment. The overlapped images in Figure 1 show the ureaplasmal intracellular location in HEp-2 cells.

#### Gentamicin invasion assay

**Figure 2:**
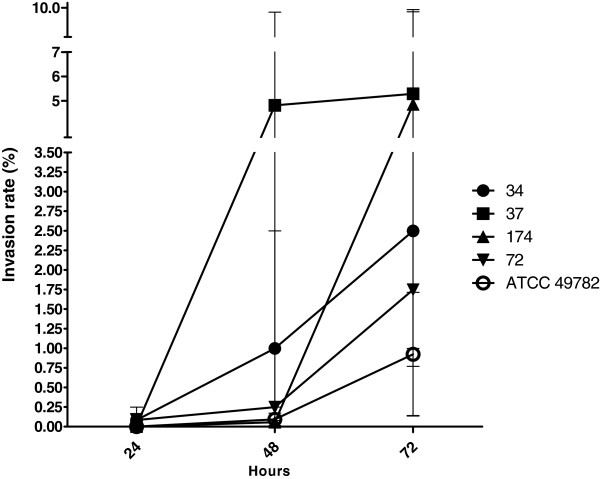
**Gentamicin invasion assay. Invasion rates of** ***U. diversum*** **strains 34, 37, 174, 72 and ATCC 49782 in Hep-2 cells after 24, 48 and 72 hours.** The studied ureaplasmas presented a higher rate of invasion between 48 and 72 hours of infection. Comparison of infection rates among strains isolated from clinical sample and standard strain ATCC 49782 (Mann–Whitney test, p-value ≤ 0.05). No statistically significant differences were found between the clinical strains and the standard strain in the study. However, a trend was observed once since invasion rates were higher when time of infection was increasing.

The studied ureaplasma presented a higher rate of invasion between 48 and 72 hours of infection (Figure 2). These differences were not stastically significant (Kruskal-Wallis, p = 0.09). No statistical differences were observed among invasion rates of studied ureaplasmas (p = 0.497). Although no statistical significance was observed, when these results were compared, isolate 37 apparently presented a higher rate of invasion. The other isolates and the reference strain of *U. diversum* apparently showed a lower rate of invasion between 24 and 48 hours of infection. Before assay, the concentration of 400 μg/ml was tested and shown to inhibit the growth of the strains tested. This figure shows the increase in the number of microorganisms internalized during infection, since gentamicin is unable to penetrate the cell. No bacterial growth was observed in uninfected cells.

### Apoptosis assays

#### Flow cytometry

**Figure 3:**
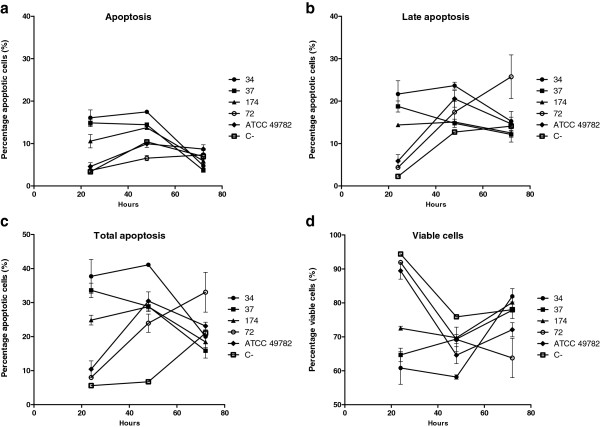
**Representation of percentage of apoptotic and viable cells obtained using flow cytometry.** HEp-2 cells infected with *Ureaplasma diversum* clinical isolates 34, 37, 174 and 72 strains and the ATCC 49782 strain. The cells were analyzed with 24, 48 and 72 hours after infection. Labeling with Annexin V (An) and Propidium Iodide (PI). Infections were performed in triplicate. The error bars represent the standard deviation from the average number of events for analysis. (C-): the negative control of uninfected cells with ureaplasmas. **a**: Percentage of apoptotic cells (An+/PI-). **b**: Percentage of cells in late apoptosis (An+/PI+). **c**: The sum of the percentage of cells undergoing apoptosis (An+/PI-) and late apoptosis (An+/PI+). **d**: Percentage of viable (An-/PI-) cells.

Figure 3 shows that all apoptotic cells in tested ureaplasma increased after 48 hours of infection. Again, only isolate 37 showed a decrease in the number of apoptotic cells for the same period of infection. After 48 hours, the number of HEp-2 apoptotic cells decreased in all isolates. This was more significant in the inoculated cells with isolates 34, 37 and 174 (Kruskal-Wallis, p <0.05). The non inoculated HEp-2 cells showed a gradual increase in the number of apoptotic cells. However, isolate 72 induced a higher number of apoptotic cells.

#### Apoptotic gene expression

**Figure 4:**
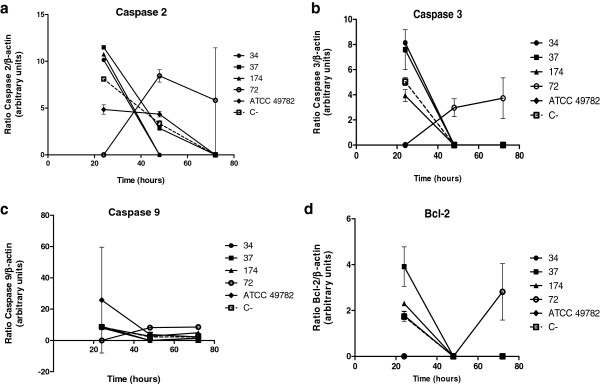
**Analysis of gene expression of some genes involved in the regulation of apoptosis, using RT-PCR.** HEp-2 cells infected with *Ureaplasma diversum* clinical isolates 34, 37, 174 and 72 strains and the ATCC 49782 strain. The cells were analyzed 24, 48 and 72 hours after infection. Infections were performed in triplicate. Error bars represent the standard deviation from the average number of events analyzed. (C-): Negative control. **a**: Gene expression of caspase 2. **b**: Gene expression of caspase 3 **c**: Gene expression of caspase 9 **d**: Gene expression of Bcl-2.

The gene for caspase 2 (Figure 4a) was expressed in HEp-2 cells inoculated with isolates 34, 37, 174 and 82; a higher expression was detected at 24 hours of infection. However, after 48 and 72 hours, the gene expression decreased to lower than in the non-inoculated HEp-2 cells (Kruskal-Wallis, p <0.05). Strain 72 obtained the highest gene expression compared to the other strains. For caspase 3 (Figure 4b), the results were similar, with the difference being that strain 82 did not induce gene expression in the entire period. No infected cells exceeded the expression of the non-infected cells.

In the initial period of infection, the ureaplasmal isolates increased caspase 9 gene expression in HEp-2 cells (Figure 4c). Observing the data of the expression of Bcl-2 gene, one can verify that after 24 hours, cells infected with two of the isolates have less Bcl-2 expression than the control. At later times, the expression is absent in all cells except for those infected with strain 72 (Figure 4d).

#### TNF-α levels

**Figure 5:**
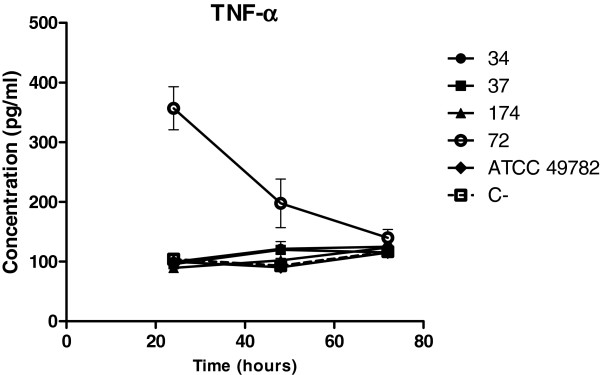
**Levels of TNF-α.** Quantification of TNF-α in culture supernatant of Hep-2 cells infected with 34, 37, 174 and 72 clinical strain isolates and ATCC 49782 strain. The cells were analyzed 24, 48 and 72 hours after infection. Infections were performed in triplicate. Error bars represent the standard deviation from the average number of events analyzed. (C-): negative control.

The amount of TNF-α produced by the HEp-2 cells infected with isolates 34, 37, 82 and 174 of ureaplasma (Figure 5) was practically the same as non infected cells. However, isolate 72, released four times as much TNF-α in HEp- 2 cells in 24hs of infection (Kruskal-Wallis, p <0.05).

## Discussion

Ureaplasmas are the major cause of genital disease of cattle, such as placentitis, granular vulvitis, infertility and abortion [23, 24]. The first isolation of these organisms was done by Taylor-Robinson et al. [25]. Ureaplasma are found in healthy animals; otherwise, they are strongly associated with respiratory and reproductive tract disorders in cattle [16]. Currently, there are few studies that correlate the virulence of *Mollicute* species and their effects on cells [26] namely species of the genus ureaplasma.

In the present study, we observed that *U. diversum* has the ability to invade HEp-2 cells, and the longer the contact time with these cells, the greater the rate of invasion. The results of the gentamicin and confocal microscopy assays confirmed the ureaplasmal intracellular location, which is consistent with previous findings mentioned by Marques et al. [22] Herein, the studied *U. diversum* isolates invaded HEp-2 cells after 1 hour of infection. Likewise, Buzinhani et al. [14] observed the invasiveness of *U. diversum* in bovine sperm. Vogl et al. [27] showed similar results with *M.gallisepticum,* which adhered and invaded non-phagocytic cells. The rate of invasion was also higher with increasing exposure time of cells to this mycoplasma. The human origin mollicutes *M. penetrans* [5] and *M. fermentans* [28] also have the ability to adhere and invade cells, remaining inside the cytoplasm, mainly in the perinuclear region [29, 30]. Krause et al. [31] reported that mutant *M. pneumoniae* lost the ability to adhere in host cells and became avirulent. The intracellular location of these bacteria protected them against the host immune response and some antibiotics [20]. The reduced genome of mollicutes renders them dependent on some nutrients that are easier to obtain in some microenvironments of the host cells. Thus their reduced metabolism may uptake amino acids, fatty acids, vitamins and cofactors [32, 29].

In the flow cytometry assay, it was observed that the number of detected apoptotic HEp-2 cells among cells infected with ureaplasmas was higher than the non-infected cells. These apoptotic HEp-2 cells were more numerous within 24 to 48 hours of infection. Harada et al. [12] showed that *U. urealyticum* also has the ability to induce apoptosis in cells. The caspase gene expression among cells infected with isolates 34, 37, 174 and 82 was also high after 24 hours of infection. The expression of Bcl-2 gene, after 24 hours, differed according to the strains used to infection. Except for infected cells with strain 72, the level of the pro-apoptotic (caspases 2, 3 and 9) and anti-apoptotic (Bcl-2) genes decreased over time (48 and 72 hours). Zhang et al. [33] observed that *Helicobacter pylori* induced a high expression of caspases 3 and 9 and low expression of Bcl-2 genes in gastric cells and concluded that the apoptosis induction was due to this bacterium. Borovsky et al. [30] reported deleterious effects on cells due to organic peroxides and free radical production by the invasion stress caused by *M. penetrans.* Cell stress can be induced by the accumulation of oxygen reactive compounds and peroxides due to degradation of fatty acids in the mycoplama metabolism [32]. These oxidative products may damage mitochondria [5]. This process in mycoplasma, coupled with attempts to acquire bacterial nucleic acids such as nutrients, can induce endonucleases and may affect DNA of the infected cells. Bendjennat et al. [11] showed that *M. penetrans* might produce endonucleases and damage DNA and RNA of host cells. These events can lead to the cytopathic effects activating the apoptotic cascade [34, 20]. All ureaplasmas process urease to obtain energy from urea and this hydrolysis produces ammonia and CO_2_ [32]. Ammonia is toxic to mammalian cells of humans and animals. Furthermore, the increase of CO_2_ can acidify the host cell microenvironment, which is also harmful to the cell [21, 35].

The number of apoptotic cells between 48 and 72 hours decreased in all experimental strains, and the number of apoptotic cells in the infected areas was lower than the control. The expression of pro-apoptotic genes between 48 and 72 hours also decreased. Rajalingam et al. [36] also observed that *Chlamydia trachomatis* in the early periods of infection induces cell apoptosis, but over longer periods of infection, these bacteria are able to inhibit apoptosis through activation of their own mechanisms of survival. Feng et al. [37] reported that certain mycoplasma infections could lead to inhibition of apoptosis. Boya et al. [9] and Massari et al. [38] also reported that *Neisseria meningitidis*, an intracellular microorganism, is able to inhibit apoptosis of host cells, probably resulting in increased cell survival and the continuous activation of B cells. With the exception of cell infection with strain 72, there was no expression of the Bcl-2 gene. This fact can be explained because there are other genes that inhibit the apoptotic cascade, which may be subsequently identified [39]. The data obtained in the present study suggests that the apoptosis may have been initially induced by some isolates in association with HEp-2 cells, but over time, there was no evidence of apoptosis in the presence of ureaplasma and HEp-2 cells. This reduction of the apoptosis rate in the infected cells to levels similar to the control cells may be directly related to the persistence of this microorganism in the intracellular environment in which it can be protected from the host immune factors.

The intial increase and then decrease in apoptosis could be related to bacterial pathogen-associated molecular pattern (PAMPS). Mollicutes possess a large number of lipoproteins, termed lipid-associated membrane proteins (LAMPs). Recognition of LAMPs by the innate immune system can trigger the production of various proinflammatory cytokines from manifold cells. On the other hand, LAMPs can directly initiate apoptotic signaling by interaction with TLR2 and TLR6 [13]. Into et al. [40] showed, after being stimulated by LAMPs, human embryonic kidney cells (HEK293) present a sequential bifurcate response: after 6 h of stimulation, LAMPs induced NF-κB activation in a dose-dependent manner, which is defined as the early event, and no cytocidal activity was shown. However, after 24 h of stimulation, apoptosis occurred. Other studies observed apoptotic activity of LAMPs [12, 41].

The release of TNF-α by HEp-2 cells infected with *U. diversum* was in similar concentrations induced by all isolates. Isolate 72, however, induced a higher amount, especially in the first 24 hours. TNF-α is a pro-inflammatory cytokine, which is involved in the activation of the apoptotic cascade via the extrinsic pathway [4]. Wu et al. [41] reported that lipoproteins *M. genitalium* are capable of increasing the expression of cytokines such as TNF-α, leading to induction of apoptosis in monocytic cells. Although isolate 72 presented a higher TNF-α in the first 24 hours, flow cytometry revealed that this isolate in this period of infection did not induce an increase in cell apoptosis. The relationship of TNF-α and absence of apoptosis is not fully understood. Arnold et al. [42] observed that *Listeria monocytogenes* and *Yersinia enterocolitica* were able to induce the release of TNF-α by HEp-2 cells, but were unable to identify virulence factors.

## Conclusions

The isolates of *U. diversum* presented differences in the studied parameters for apoptosis. Although Bcl-2 family members are often controlled via expression levels and the ratio of pro- and anti-apoptotic family members mediates the apoptotic susceptibility of cells, caspase enzyme activity is primarily regulated at the post-translational level. Perhaps the use of post-transcriptional methodologies is more credible for analysis. It was also observed that the amount of microorganisms was not proportional to the induction of apoptosis in HEp-2 cells. This may explain, in part, the findings of these bacteria in healthy or deceased bovines. Therefore the indication of variations among ureaplasmas originating from bovine confirmed the genotyping data, but still do not allow for identifying a profile of a candidate for their pathogenesis. As in many studies with other *Mollicutes*, ureaplasma may be also included as a diverse group with important genotypic and phenotypic variations also making them variable bacteria in bovines.

## Methods

### *Ureaplasma diversum*and cell lines

Four isolates of *U. diversum* and the strain, ATCC 49782, were studied. The clinical isolates (34, 37, 72, 82 and 174 isolates) were previously recovered from vulvovaginal swabs of cows with granulomatous disease from a dairy farm in the southwestern region of Bahia, Brazil. The isolates were characterized by their growth and identified by their species-specific PCR methodology [43].

HEp-2 cell lines were previously certified to be free of *Mollicutes* by culture and PCR [44]. The cells were cultured in 5% CO_2_ at 37°C in Minimum Essential Medium (MEM) with 2 mM L-glutamine and Earle’s balanced salts, supplemented with 10% fetal calf serum (Cult Lab, São Paulo, Brazil). Twenty-four hours prior to ureaplasmal infection, HEp-2 cell monolayers were established for 10–20% confluence, in 24-well micro plates (TPP – Switzerland), with one ml of MEM medium (Cult Lab, São Paulo, Brazil).

### Labelling and quantification of ureaplasma cells

The ureaplasmas were first cultured in 2 ml of ureaplasma broth (UB) at 37°C and grown to a culture of 50 ml. In a logarithmic growth phase, the culture was centrifuged at 20,600 g for 30 minutes at 25°C. The pellets were divided into three vials. The first was homogenized by washing twice with Phosphate Buffered Saline (PBS) and incubated with carbocyanine dye solution (Vybrant™ Dil cell-labeling solution-Dil, V-22885, Molecular Probe, Eugene, Oregon, USA) to confocal laser scanning microscopy assay. The second and third vials were not labeled and were used for gentamicin invasion and apoptosis assays, respectively. The number of ureaplasma cells inoculated in each assay was determined by 10-fold dilution in UB medium and expressed as Color Changing Units/ml (CCU/ml).

### Inoculation of ureaplasma in HEp-2 cells

The methodology was based on Baseman et al. [29]. Cells at a confluence of approximately 60 to 70% and 10^6^ cells/glass slides were selected. These cells were initially washed with PBS and inoculated with 10^5^ to 10^7^ of labeled mycoplasmas contained in one ml of MEM with 2% bovine fetal serum. The sets of inoculated cells were incubated at 37°C in 5% CO_2_ atmosphere and kept for 24, 48 and 72 hours. After each time of infection, the bacterial suspension was gently removed and each well was washed three times with PBS. Then the remaining cells were fixed with 3.7% formaldehyde in PBS for 30 minutes at room temperature, washed three times with PBS and treated with 0.05% Triton X-100 for 10 minutes. This procedure prepares the cells to be labeled.

### Invasion assays

#### Confocal laser scanning microscopy

For cell cytoskeleton visualization, the cells were stained for 30 minutes at 37°C with phalloidin associated with fluoresceine-isothiocyanate (Sigma) diluted at 1:200. This fluorochrome was removed with three washings of PBS. Then, the cells were treated with RNAase (10 mg/ml) for 30 minutes. The nuclei were stained with TO-PRO-3 (Molecular Probes, dilution 1:500). The HEp-2 cells and ureaplasma were mounted with antifading solution (Vecta Shield, Vector Laboratories, Burlingame, CA, USA) on histological slides. The infected and non-infected HEp-2 cells were observed under Confocal Laser Scanning Microscope - CLSM (Carl Zeiss LSM 510, Germany, equipped with Argon laser, 488 nm, and 2 helium/neon 543 nm wavelengths) to visualize the luminescence of fluochromes. This procedure was used to detect ureplasmal positioning in HEp-2 after infection.

#### Gentamicin invasion assay

The gentamicin invasion assay was performed to determine the invasion rate of viable ureaplasma inside the HEp-2 cells. The methodology used was described by Yavlovich et al. [45]. The amount of 10^4^ HEp-2 cells per well were initially seeded in 24-well microplates. After 24 hours of incubation at 37°C in 5% CO_2_, the cell cultures were inoculated with 10^5^ to 10^7^ ureaplasmas (CCU/ml). The infected cells were incubated for 24, 48 and 72 hours, washed three times with PBS and incubated for an additional three hours in MEM (1 ml/well) containing 400 μg/ml of gentamicin to eliminate the non internalized ureaplasma. The antibiotic solution was removed, and the infected cells were trypsinized and sub-cultured in UB broth. The remaining ureaplasmas were quantified by CCU methodology and performed in triplicate. These CCU were compared with the initial values of ureaplasmal suspensions. The same procedure was performed with uninfected cells.

### Apoptosis assays

#### Flow cytometry

The percentage of HEp-2 apoptotic cells inoculated with *U. diversum* was measured by flow cytometry BD FACS Calibur™ [4]. Cells were previously labeled with fluorochromes Annexin V and propidium iodide (PI) both in the kit Alexa Fluor® 488 (Molecular Probes). Fluorochromes are those suitable for apoptosis analysis by flow cytometry [46, 47]. In apoptotic cells, phosphatidylserine (PS) is translocated from the inner to the outer leaflet of the plasma membrane, thus exposing PS to the external cellular environment. In leukocyte apoptosis, PS on the outer surface of the cell marks the cell for recognition and phagocytosis by macrophages. The human anticoagulant, annexin V, is a 35–36 kDa Ca^2+^-dependent phospholipid-binding protein, which has a high affinity for PS. Annexin V labeled with a fluorophore or biotin can identify apoptotic cells by binding to PS exposed on the outer leaflet. The FITC Annexin V/Dead Cell Apoptosis Kit with FITC annexin V and PI for flow cytometry provides a convenient assay for apoptosis. Cell populations were separated into quadrants determined by individual controls containing PI, annexin V and cell only samples, which produce different fluorescence intensity gate settings for the experimental samples. The percentage of apoptotic cells (Annexin positive/PI negative), late apoptosis (Annexin positive/PI positive), total apoptosis (sum of apoptotic cells plus late apoptosis) and viable cells (Annexin negative/PI negative) were analyzed. Samples were analyzed using a BD FACS Calibur™, which measured fluorescence emission at 530 nm and 575 nm for PI and annexin V, respectively. Data were expressed as percentages of populations containing 1 × 10^5^ cells. For each detection, the procedure was repeated three times. Unifected cells and Cells incubed with hydrogen peroxide (1 mM) were used as negative and positive control populations to set the gates. Cells stained only with FITC annexin V and only with PI were also used for fluorescence calibration.

#### Apoptotic gene expression

In order to evaluate the genes of caspases 2, 3 and 9 and anti-apoptotic gene Bcl-2 involved in apoptosis, semi-quantitative RT-PCR (Polymerase Chain Reaction with Reverse Transcriptase) was used by reverse transcription of mRNA total and polymerase chain reaction. The cDNA was synthesized from total mRNA using oligodT. Specific amplification of cDNA from caspases 2, 3 and 9 and Bcl-2 was performed by PCR. The primers and reaction parameters for caspases were performed according to Fortunato et al. [48] and Bcl-2 according to Zhang et al. [33]. The amplified cDNA was electrophoresed on 2% agarose gels containing ethidium bromide, and quantities were analyzed by densitometry using ImageJ software (the Research Service Branch of the National Institute of Health, Bethesda, MD, USA). The relative expression of each gene was normalized to the intensity of a housekeeping gene, β-actin. The expression level of each gene is reported as a ratio relative to the level of normalized expression in a control sample.

#### TNF-α measures

The sets of HEp-2 cells inoculated or not with ureaplasma were incubated at 37°C in 5% CO_2_ atmosphere for 24, 48 and 72 hours. The supernatants of these sets were removed from the wells of microplates and measured for cytokines TNF-α using the ELISA methodology according to the manufacturer instructions (Bioscience, San Diego, CA).

### Statistical analysis

The differences between infected and non-infected cells were analyzed using One-way ANOVA followed by Kruskal-Wallis test and Dunn’s post-test. Analyses were performed using GraphPad Prism® software (version 5.0, GraphPad Software, San Diego, CA, USA). Statistical differences were considered significant at p values <0.05 in a confidence interval of 95%.
